# New Books

**Published:** 2005-04

**Comparative Genomics: RECOMB 2004 International Workshop, RCG 2004**

Jens Lagergren, ed.

New York:Springer-Verlag, 2005. 133 pp. ISBN: 3-540-24455-7, $58

**DNA Methylation and Cancer Therapy**

Moshe Szyf, ed.

New York:Springer-Verlag, 2005. 255 pp. ISBN: 0-306-47848-X, $139

**Environmental Chemistry: Chemistry of Major Environmental Cycles**

Teh Fu Yen

Hackensack, NJ:World Scientific Publishing Co., 2005. 650 pp. ISBN: 1-86094-474-4, $76

**Essentials of Medical Geology: Impacts of the Natural Environment on Public Health**

Olle Selinus

Burlington, MA:Elsevier, 2005. 832 pp. ISBN: 0-12-636341-2, $99.95

**Evaluation of Enzyme Inhibitors in Drug Discovery: A Guide for Medicinal Chemists and Pharmacologists**

Robert A. Copeland

Hoboken, NJ:John Wiley & Sons, Inc., 2005. 296 pp. ISBN: 0-471-68696-4, $84.95

**Fed Up! Winning the War Against Childhood Obesity**

Susan Okie

Washington, DC:National Academies Press, 2005. 336 pp. ISBN: 0-309-09310-4, $27.95

**Global Intelligence and Human Development: Toward an Ecology of Global Learning**

Mihai I. Spariosu

Cambridge, MA:MIT Press, 2005. 296 pp. ISBN: 0-262-69316-X, $23

**Global Warming: A Very Short Introduction**

Mark Maslin

New York:Oxford University Press, 2005. 184 pp. ISBN: 0-19-284097-5, $9.95

**Handbook of RNA Biochemistry, 2 Vols.**

Roland Karl Hartmann, Albrecht Bindereif, Astrid Schön, Eric Westhof, eds.

Hoboken, NJ:John Wiley & Sons, Inc., 2005. 932 pp. ISBN: 3-527-30826-1, $385

**London’s Environment: Prospects for a Sustainable World City**

Julian Hunt, ed.

Hackensack, NJ:World Scientific Publishing Co., 2005. 350 pp. ISBN: 1-86094-486-8, $80

**Natural Arsenic in Groundwater**

J. Bundschuh, P. Bhattacharya, D. Chandrasekharam

Leiden, The Netherlands:A.A. Balkema Publishers, 2005. 356 pp. ISBN: 4-1536-700-X, $109

**Occupational and Residential Exposure Assessment for Pesticides**

Claire Franklin, John Worgan

Hoboken, NJ:John Wiley & Sons, Inc., 2005. 438 pp. ISBN: 0-471-48989-1, $198

**Paths to a Green World: The Political Economy of the Global Environment**

Jennifer Clapp, Peter Dauvergne

Cambridge, MA:MIT Press, 2005. 336 pp. ISBN: 0-262-03329-1, $62

**Reviews of Environmental Contamination and Toxicology**

George W. Ware

New York:Springer-Verlag, 2005. 243 pp. ISBN: 0-387-22398-3, $129

**RNA Methodologies**

Robert Farrell Jr.

Burlington, MA:Elsevier, 2005. 688 pp. ISBN: 0-12-249696-5, $99.95

**Science and Technology in the National Interest: Ensuring the Best Presidential and Federal Advisory Committee Science and Technology Appointments**

Committee on Ensuring the Best Presidential and Federal Advisory Committee Science and Technology Appointments

Washington, DC:National Academies Press, 2005. 224 pp. ISBN: 0-309-09297-3, $45.50

**Street Science: Community Knowledge and Environmental Health Justice**

Jason Corburn

Cambridge, MA:MIT Press, 2005. 304 pp. ISBN: 0-262-03333-X, $60

**The State and the Global Ecological Crisis**

John Barry, Robyn Eckersley, eds.

Cambridge, MA:MIT Press, 2005. 368 pp. ISBN: 0-262-02581-7, $67

